# Ramsay-Hunt Syndrome in Patients Undergoing Dose-Dense Chemotherapy in the Perioperative Period of Breast Cancer: Two Case Reports

**DOI:** 10.7759/cureus.100372

**Published:** 2025-12-29

**Authors:** Emi Kanaya, Koshi Matsui, Ameri Urasaki, Mutsuki Furukawa, Shiho Nagasawa, Misato Araki, Shinichi Sekine, Tsutomu Fujii

**Affiliations:** 1 Department of Surgery and Science, Faculty of Medicine, Academic Assembly, University of Toyama, Toyama, JPN

**Keywords:** breast cancer, chemotherapy, lymphopenia, pneumocystis pneumonia (pcp), ramsay hunt syndrome

## Abstract

Dose-dense doxorubicin and cyclophosphamide followed by dose-dense paclitaxel (ddAC-ddPTX therapy) is a treatment strategy that shortens intervals between chemotherapy cycles and has demonstrated efficacy in improving disease-free survival (DFS) and overall survival (OS). Moreover, this strategy is recommended by the National Comprehensive Cancer Network (NCCN) guidelines. However, adverse events, such as lymphopenia, which can lead to opportunistic infections such as pneumocystis pneumonia, have been reported, thus necessitating appropriate countermeasures. Herein, we report two rare cases of Ramsay-Hunt syndrome associated with lymphopenia during ddAC-ddPTX therapy.

## Introduction

Dose-dense doxorubicin and cyclophosphamide followed by dose-dense paclitaxel (ddAC-ddPTX) is a standard perioperative treatment strategy that shortens intervals between chemotherapy cycles. This regimen has demonstrated efficacy in improving disease-free survival (DFS) and overall survival (OS) in breast cancer patients and is widely recommended in clinical guidelines, including those of the National Comprehensive Cancer Network (NCCN).

However, the intensive nature of this regimen frequently leads to severe adverse events. Beyond standard myelosuppression (neutropenia), chemotherapy significantly modulates the host immune system [1]. Specifically, dose-dense regimens are associated with a high incidence of lymphopenia; reports indicate that significant lymphocyte depletion occurs in over 60% of treated patients [2]. While neutropenia management is well-established using granulocyte colony-stimulating factor (G-CSF), lymphopenia, which predisposes patients to viral and fungal infections, often receives less attention.

Although hematologic malignancies are traditionally considered the primary risk group for varicella-zoster virus (VZV) reactivation, recent epidemiological data suggest that patients with solid tumors also face a significantly elevated risk, approximately double that of the general population [3]. Ramsay Hunt syndrome (RHS), caused by VZV reactivation in the geniculate ganglion, is a particularly severe complication. Compared to Bell's palsy, RHS is associated with a poorer prognosis for facial nerve recovery and a higher risk of permanent sequelae [4].

While *Pneumocystis jirovecii *pneumonia (PCP) is a well-recognized complication of lymphopenia during dose-dense chemotherapy [5], reports of RHS in this context are extremely rare. Herein, we report two cases of RHS associated with severe lymphopenia during ddAC-ddPTX therapy, discussing the potential mechanisms, management strategies, and the importance of early diagnosis.

## Case presentation

Case 1

A 57-year-old postmenopausal woman presented with left ear pain and facial nerve paralysis. She was diagnosed with luminal-HER2-positive right breast cancer (cT1cN0M0, cStage I). Regarding her medical history, she had no known history of prior varicella infection or herpes zoster, and her varicella-zoster vaccination status was not documented. Furthermore, she had no pre-existing immunosuppressive comorbidities, such as autoimmune diseases or diabetes mellitus, and her nutritional status and body mass index were within normal limits.

**Figure 1:**
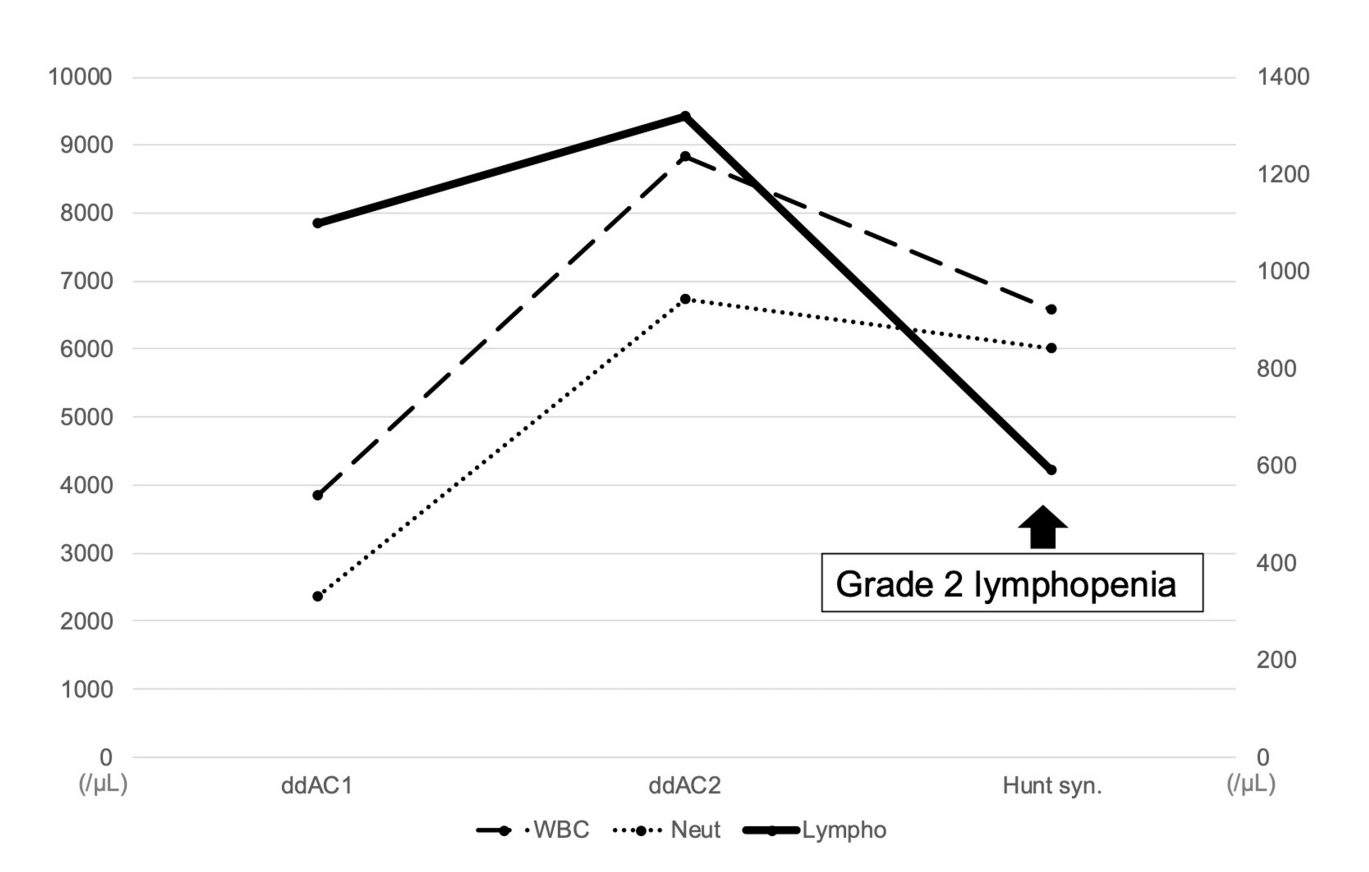
Trends in WBC, neutrophil, and lymphocyte counts in Patient 1.

The patient underwent a partial mastectomy with a sentinel lymph node biopsy. After postoperative radiotherapy, adjuvant chemotherapy with ddAC followed by ddPTX + trastuzumab was initiated. On the 10th day after completing the second cycle of ddAC, she developed left ear pain, facial paralysis, and dizziness, which prompted her to visit our emergency department. Physical examination revealed taste disturbances, erythema in the left external auditory canal, swelling of the left anterior auricle, and facial nerve paralysis (Yanagihara score of 6/40). The laboratory findings indicated grade 2 lymphopenia with a lymphocyte count of 590/μL (Figure 1).

Based on these findings, the patient was diagnosed with complete left-sided RHS. She was admitted to the otolaryngology department for inpatient management, and treatment was initiated with intravenous acyclovir (750 mg/day) and intravenous dexamethasone (6.6 mg/day). Following clinical stabilization, she was discharged on the ninth day of hospitalization. Although adjuvant therapy was resumed three months later, the ddAC-ddPTX regimen was discontinued; instead, treatment was continued with trastuzumab monotherapy to avoid further chemotherapy-induced immunosuppression. While the facial paralysis showed moderate improvement, residual symptoms persisted (Yanagihara score: 22/40). The patient has remained recurrence-free for six years since the initial surgery.

Case 2

A 69-year-old postmenopausal woman was diagnosed with triple-negative breast cancer (cT2N0M0, Stage II) and was scheduled to receive neoadjuvant chemotherapy. Regarding her medical history, she had no known history of prior varicella infection or herpes zoster, and her varicella-zoster vaccination status was not documented. She had no pre-existing immunosuppressive comorbidities, such as autoimmune diseases or diabetes mellitus, and there were no other obvious host-related risk factors for immunosuppression.

**Figure 2:**
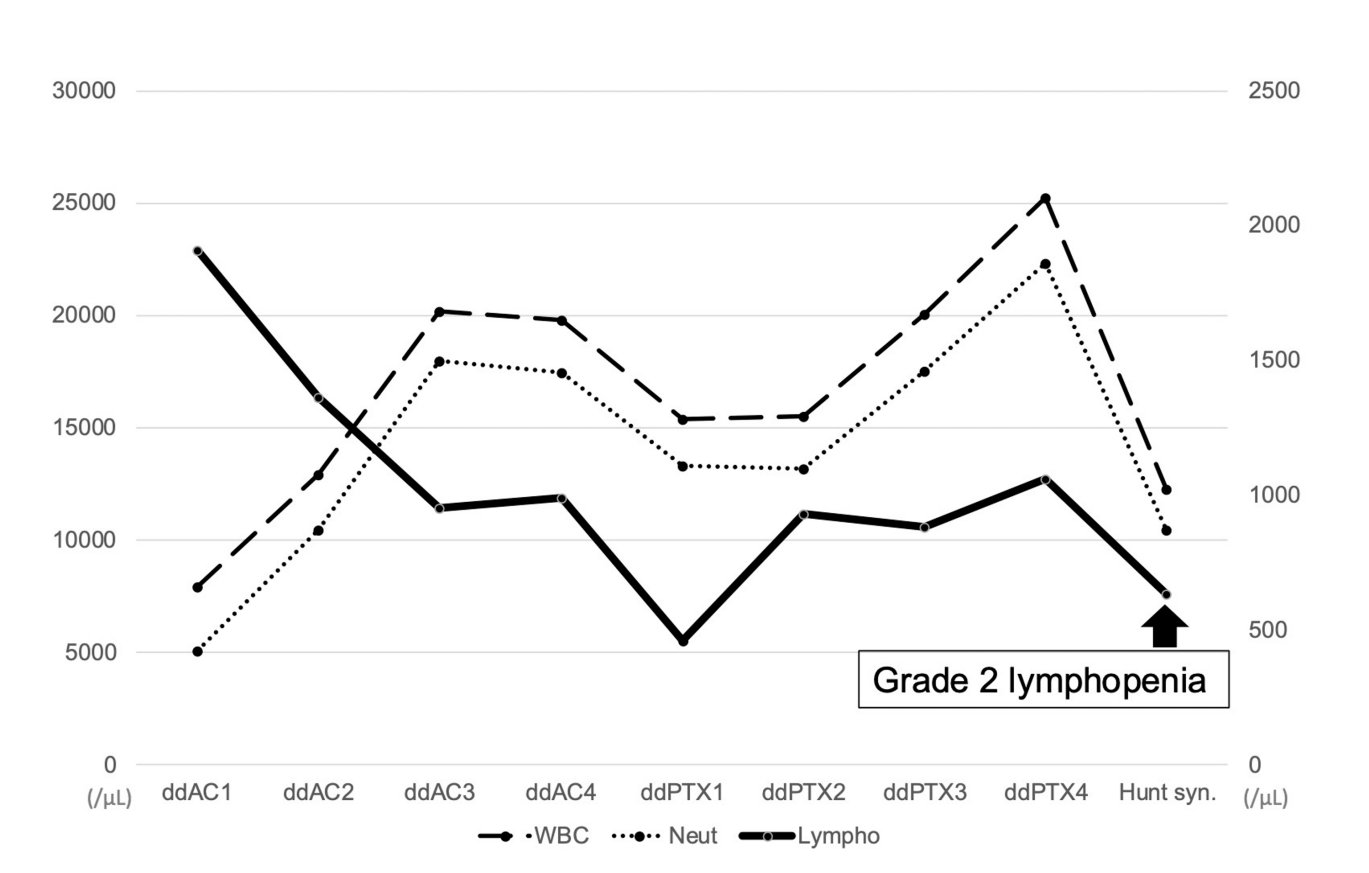
Trends in WBC, neutrophil, and lymphocyte counts in Patient 2.

She received neoadjuvant ddAC-ddPTX therapy. On the 10th day after the third cycle of ddPTX, the patient presented to our emergency department with acute-onset right ear pain, facial nerve paralysis, and dizziness. Physical examination revealed erythema of the right auricle and external auditory canal, along with right facial nerve palsy (Yanagihara score of 12/40). Laboratory investigations indicated grade 2 lymphopenia, with an absolute lymphocyte count of 630/μL (Figure 2).

Based on these findings, the patient was diagnosed with complete right-sided RHS. She was admitted to the otolaryngology department for inpatient treatment, and intravenous acyclovir (750 mg/day) and intravenous dexamethasone (6.6 mg/day) were started. She was discharged on the eighth day of hospitalization following clinical improvement. After the onset of RHS, the ddAC-ddPTX regimen was discontinued, and the patient subsequently underwent total mastectomy with sentinel lymph node biopsy three months later, achieving a pathological complete response (pCR). Although her facial nerve palsy improved, mild residual symptoms persisted (Yanagihara score: 24/40). The patient has remained recurrence-free for five years since surgery.

## Discussion

RHS is caused by the reactivation of varicella-zoster virus (VZV) in the geniculate ganglion of the facial nerve, leading to herpes zoster oticus and inflammatory damage to the facial nerve within the facial canal. Its clinical manifestations are diverse and severe, including not only facial nerve paralysis but also vesicular eruptions in the auricle or external auditory canal, vertigo, hearing loss, tinnitus, and taste disturbance affecting the anterior two-thirds of the tongue. Compared to Bell’s palsy, RHS is associated with a significantly poorer prognosis. While the natural recovery rate for Bell’s palsy exceeds 70%, complete recovery in RHS is reported to be only approximately 30-70% [4]. Treatment outcomes are heavily dependent on the timing of intervention; Murakami et al. demonstrated that early administration of corticosteroids combined with antiviral agents significantly improves nerve recovery [6].

From a clinical standpoint, several key points are useful in differentiating RHS from idiopathic facial palsy (Bell’s palsy). In RHS, severe ear pain and vesicular eruptions around the auricle or within the external auditory canal are characteristic findings, whereas such cutaneous lesions are absent in Bell’s palsy. In addition, vestibulocochlear symptoms, such as vertigo, tinnitus, and hearing loss, are more frequently observed and often more pronounced in RHS. Multiple cranial nerve involvement, as reflected by concomitant taste disturbance in the anterior two-thirds of the tongue, also favors a diagnosis of RHS. In both of our cases, the combination of severe ear pain, typical vesicular eruptions involving the auricle or external auditory canal, vestibular symptoms, and concomitant taste disturbance was considered incompatible with idiopathic Bell’s palsy and strongly supportive of a diagnosis of RHS. Therefore, in breast cancer patients receiving dose-dense chemotherapy who present with acute facial paralysis accompanied by ear pain or auricular skin changes, clinicians should maintain a high index of suspicion for RHS rather than assuming idiopathic Bell’s palsy.

In the present cases, the RHS onset coincided with the nadir of lymphopenia induced by dose-dense chemotherapy. These observations highlight that, given the risk of permanent neurological sequelae, early differentiation from Bell’s palsy is particularly critical in the oncology setting.

The severe lymphopenia observed in our cases was likely driven by a synergistic immunosuppressive mechanism involving both cytotoxic myelosuppression and corticosteroid administration. Chemotherapy targets rapidly dividing cells and modulates the host immune system [1]. Specifically, dose-dense regimens (ddAC-ddPTX) shorten the bone marrow recovery interval, leading to cumulative depletion of lymphocytes; Tolaney et al. reported that severe lymphopenia (grades 3-4) occurred in 63% of patients receiving dose-dense regimens [2]. Verma et al. further reported that, following breast cancer chemotherapy, the recovery of CD4+ T lymphocytes, which are pivotal for VZV control, is significantly delayed and remains impaired long after treatment cessation [7]. Furthermore, the concomitant use of dexamethasone for antiemetic prophylaxis exacerbates this impairment. Corticosteroids selectively deplete T cells and suppress IL-2 production, causing a shift from a Th1-dominant immune response to a Th2-dominant response [8]. Even low-dose steroids can increase the risk of viral reactivation, as seen in other autoimmune conditions [9]. This synergistic immunosuppressive effect creates a significant window of immunological vulnerability.

Although hematologic malignancies are traditionally associated with the highest risk of VZV reactivation, patients with solid tumors also face a significantly elevated risk. Habel et al. reported that the incidence of herpes zoster in patients with solid tumors is approximately double that of the general population [3]. While prophylactic trimethoprim-sulfamethoxazole (TMP-SMX) is routinely administered for PCP in such high-risk groups [5,10], it offers no protection against viral reactivation. Several cases of PCP during dose-dense chemotherapy have been reported [11,12], but these cases highlight that severe lymphopenia in solid tumor patients carries a broader spectrum of infectious risks, including VZV, than previously emphasized.

To mitigate these risks, our institution has adopted strategies to minimize steroid exposure, such as limiting dexamethasone to chemotherapy days and utilizing olanzapine as an alternative antiemetic agent. However, even with these measures, VZV reactivation can still occur. Clinicians must remain vigilant for RHS in breast cancer patients undergoing dose-dense chemotherapy, particularly when lymphocyte counts are markedly suppressed. Since current guidelines, such as those from the NCCN, do not routinely recommend antiviral prophylaxis for patients with solid tumors [13], patient education is paramount.

In both of our cases, prophylactic antiviral therapy was not administered when resuming or initiating subsequent anticancer treatment. This decision was based on the discontinuation of the dose-dense chemotherapy regimen (ddAC-ddPTX), the transition to trastuzumab monotherapy or surgery alone, and the lack of guideline support for routine antiviral prophylaxis in this setting. Patients should be explicitly instructed to report symptoms such as ear pain, dizziness, or vesicular eruptions immediately. Prompt initiation of antiviral therapy is essential to maximize the chances of functional nerve recovery.

## Conclusions

We reported two rare cases of RHS associated with lymphopenia during ddAC-ddPTX therapy. In addition to PCP, clinicians should remain vigilant for RHS as a potential complication in patients with significant lymphopenia. Preventive measures and early treatment are essential for managing risks and minimizing long-term impacts on patients' quality of life.
